# Relationships between biological age, distance from aquatic habitats and pyrethroid resistance status of *Anopheles funestus* mosquitoes in south-eastern Tanzania

**DOI:** 10.1186/s12936-022-04389-y

**Published:** 2022-12-02

**Authors:** Polius G. Pinda, Dickson S. Msaky, Letus L. Muyaga, Issa H. Mshani, Rukiyah M. Njalambaha, Japhet Kihonda, Hamis Bwanaly, Halfan S. Ngowo, Emmanuel W. Kaindoa, Lizette L. Koekemoer, Fredros O. Okumu

**Affiliations:** 1 Environmental Health and Ecological Sciences Department, Ifakara Health Institute, Morogoro, United Republic of Tanzania; 2 School of Life Sciences and Biotechnology, Nelson Mandela African Institution of Science and Technology, Arusha, United Republic of Tanzania; 3 School of Public Health, University of the Witwatersrand, Johannesburg, South Africa; 4 Institute of Biodiversity, Animal Health and Comparative Medicine, University of Glasgow, Glasgow, UK; 5 Wits Research Institute for Malaria, Faculty of Health Sciences, Centre for Emerging Zoonotic and Parasitic Diseases, University of the Witwatersrand, National Institute for Communicable Diseases, Johannesburg, South Africa

**Keywords:** Insecticide resistance, Mosquito age, Parity, Age grading, *Anopheles funestus*, Piperonyl butoxide, Malaria, Aquatic habitats

## Abstract

**Background:**

Malaria transmission can be highly heterogeneous between and within localities, and is influenced by factors such as survival and biting frequencies of *Anopheles* mosquitoes. This study investigated the relationships between the biological age, distance from aquatic habitats and pyrethroid resistance status of *Anopheles funestus* mosquitoes, which currently dominate malaria transmission in south-east Tanzania. The study also examined how such relationships may influence malaria transmission and control.

**Methods:**

Female *An. funestus* were collected in houses located 50–100 m, 150–200 m or over 200 m from the nearest known aquatic habitats. The mosquitoes were exposed to 1×, 5× and 10× the diagnostic doses of deltamethrin or permethrin, or to the synergist, piperonyl butoxide (PBO) followed by the pyrethroids, then monitored for 24 h-mortality. Ovaries of exposed and non-exposed mosquitoes were dissected to assess parity as a proxy for biological age. Adults emerging from larval collections in the same villages were tested against the same insecticides at 3–5, 8–11 or 17–20 days old.

**Findings:**

Mosquitoes collected nearest to the aquatic habitats (50-100 m) had the lowest mortalities compared to other distances, with a maximum of 51% mortality at 10× permethrin. For the age-synchronized mosquitoes collected as larvae, the insecticide-induced mortality assessed at both the diagnostic and multiplicative doses (1×, 5× and 10×) increased with mosquito age. The highest mortalities at 1× doses were observed among the oldest mosquitoes (17–20 days). At 10× doses, mortalities were 99% (permethrin) and 76% (deltamethrin) among 8–11 day-olds compared to 80% (permethrin) and 58% (deltamethrin) among 3–5 day-olds. Pre-exposure to PBO increased the potency of both pyrethroids. The proportion of parous females was highest among mosquitoes collected farthest from the habitats.

**Conclusion:**

In this specific setting, older *An. funestus* and those collected farthest from the aquatic habitats (near the centre of the village) were more susceptible to pyrethroids than the younger ones and those caught nearest to the habitats. These findings suggest that pyrethroid-based interventions may remain at least moderately effective despite widespread pyrethroid-resistance, by killing the older, less-resistant and potentially-infective mosquitoes. Further studies should investigate how and whether these observations could be exploited to optimize malaria control in different settings.

## Background

The risk of malaria transmission in endemic communities is influenced by multiple environmental and biological factors, often leading to a heterogeneous pattern, which can be further influenced by intervention [1–4]. These factors include the distribution of mosquito breeding habitats and human dwellings [5–7], as well as mosquito-related factors such as blood-feeding behaviours, dispersal range and non-random host selection [8–11]. When human settlements are close to the breeding sites, the range at which mosquitoes can disperse is limited [12], and vector densities tend to be highest where the settlements are most concentrated [11]. These variations can occur at all levels of malaria endemicity, and at different geographical scales. However, they tend to be greater in low-transmission compared to high-transmission areas and can be significant over fine geographic scales such as between or within villages [1].

The landscape-level variations, especially as influenced by non-random mosquito-biting frequencies, have been elucidated broadly both in situ and *in silico*, and are known to have significant epidemiological implications [1, 9, 13]. Understanding these variations of malaria transmission risk can enable more strategic approaches and better resource allocation for malaria control, even though the actual impact of such targeting remains unclear and poorly understood [14]. It is well known that female *Anopheles* mosquitoes can travel for varied distances [12, 15], but final aggregations of these vectors tend to be highest where human densities and household occupancy are greatest [11]. Also, while mosquito densities tend to be highest near their aquatic habitats, it is the females found far from their larval habitations that tend to be older and more likely to be infected with malaria parasites [7]. Furthermore, the infectivity of wild-caught mosquitoes depends on their biological age and the density of the parasite at ingestion [16]. This implies differential infectivity since malaria vectors generally require more than ten days to ensure complete parasite development and maturation [17].

Beyond the environmental factors and their influence on mosquito dispersal, experimental observations have also shown that the susceptibility of mosquitoes to insecticides can increase with age; and that older mosquitoes are generally more likely to be killed than younger ones [18–20]. Moreover, resistance in major malaria vectors can vary seasonally and spatially, in some cases at fine geographical scales [21, 22]. The observations may also have direct implications on the effectiveness of the insecticidal interventions commonly used against malaria vectors, namely insecticide-treated nets (ITNs) and indoor residual spraying (IRS). Unfortunately, there is currently limited research on how these interactions play out in communities such as rural south-eastern Tanzania, where ITNs have been widely used for more than two decades; and malaria transmission risk has become highly fragmented on a spatial scale, with greater than 10 fold differences in transmission intensity over short distances less than 50 km [23–27]. In these areas, the dominant vector species is *An. funestus*, which mediates over 80% of all malaria transmission events [25, 26]. In particular, there has not been any detailed analysis that integrates observations of the environmental factors (e.g. distance from habitats) and biological factors (e.g. mosquito infectivity and dispersal rates) with intervention-related factors (e.g. insecticide resistance levels in the dominant malaria vectors); and how such correlations may influence disease transmission and control.

This study, therefore, assessed, at fine-scale, the interplay between key biological and environmental factors that influence malaria transmission by *An. funestus* in rural south-eastern Tanzania. The key factors investigated were: (a) age of *An. funestus* mosquitoes, (b) the susceptibility of these mosquitoes to pyrethroid insecticides, and (c) the spatial distances from nearest aquatic habitats.

## Methods

### Study site

**Fig. 1:**
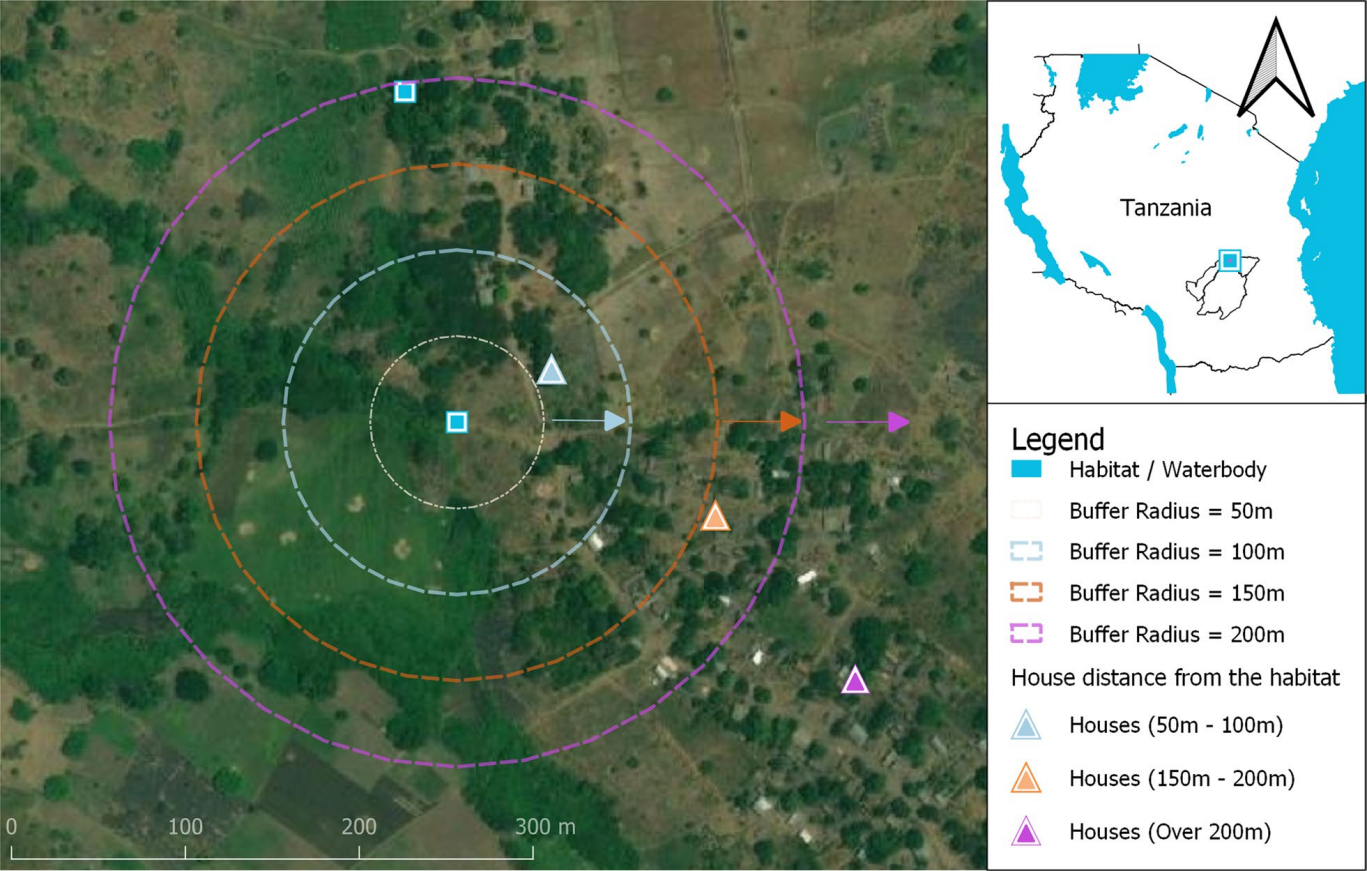
Map of the study area as a representative for adult mosquito collections at different distances from aquatic habitats

Adult female *An. funestus* mosquitoes were collected from the spatially isolated hamlet in Ikwambi village in Kilombero district, south-eastern Tanzania (location: 7.98033°S, 36.81701°E; altitude: 400 m above the sea level; annual rainfall: 1200–1800 mm; temperatures: 20–33 °C) [28] (Fig. 1). *Anopheles funestus* dominates this village in both densities and malaria transmission activity. The collections were done in the dry months from August to early December 2020.

### Mosquito collections

Initial surveys were undertaken to identify and mark all possible aquatic habitats of *An. funestus*, based on attributes and methods previously described by Nambunga et al. [29]. The habitats generally had clear waters, were formed by the river stream, had emergent vegetation and remained with water even during the dry season. The distance from confirmed habitats of *An. funestus* to human habitations were calculated based on geo-locations data obtained using a handled GPS receiver (eTrex, Vista, Garmin, USA). Three distance ranges were defined from the aquatic habitat as follows; (a) 50–100 m (edge of the village), (b) 150–200 m (intermediate distance), and (c) over 200 m (at the centre of the village) (Fig. 1).

Centre for Disease Control and Prevention (CDC) light traps placed beside volunteer-occupied bed nets were used to collect host-seeking female mosquitoes indoors from 18:00 to 07:00 h each night. The collections were grouped by distance and acclimatized for 24 h in an insectary where they were supplied with 10% glucose, and only the survivors were used for subsequent tests. Additional collections were done using volunteer-occupied miniaturized double net (DN-Mini) traps placed near human dwellings [30]. The identification of the collected mosquitoes was done using dichotomous keys of African *Anopheles* [31].

Besides the adult collections, *An. funestus* larvae were also collected using procedures described by Nambunga et al., [29] and transported in their natural water for onward rearing in the insectary (at 80 ± 10% relative humidity and 27 ± 2 °C) at the Ifakara Health Institute vector biology laboratory, the VectorSphere. Tetramin® fish food (Tetra GmbH, Melle, Germany) was provided to supplement the aquatic diet, and the pupae collected were placed in separate cages supplied with 10% glucose. The emergent, mosquitoes were used to assess variations of susceptibility in three age groups, as follows; (i) 3–5 days, (ii) 8–11 days and (iii) 17–20 days.

### Insecticide resistance bioassays

Two pyrethroids (deltamethrin and permethrin) were evaluated using WHO guidelines [32], starting with standard diagnostic insecticide doses (1×) for phenotypic resistance evaluation, followed by 5× and 10× the diagnostic doses to assess intensities of resistance. Since malaria vectors in the area are resistant to pyrethroids, additional tests were done using the synergist, piperonyl butoxide (PBO), to investigate the role of cytochrome P450 enzymes, and the potency of PBO in reversing resistance [21, 22].

Each assay consisted of six replicates with 20 individual mosquitoes per replicate, totalling 120 mosquitoes per bioassay. In the first round of bioassays (using adults collected from houses), the tests were done separately for each distance range from aquatic habitats (50–100 m, 150–200 m and over 200 m); and in the second round (using age-synchronized adults collected as larvae) the tests were done separately for the different age classes (3–5, 8–11 and 17–20 days). Mosquitoes were exposed to the insecticides for 60 min and moved to non-insecticidal holding tubes with 10% glucose, then monitored for 24 h post-exposure mortalities. Since the mosquitoes were resistant to the diagnostic concentrations of both pyrethroids, the additional tests for resistance intensity were completed as prescribed by WHO [32].

For synergy tests with PBO four cohorts of mosquitoes were exposed to: (i) deltamethrin or permethrin only at diagnostic concentration, (ii) 4% PBO followed by diagnostic dose of deltamethrin or permethrin, (iii) 4% PBO only, or (iv) silicone oil coated papers (control group). Each test was repeated three times, and the mosquitoes were provided 10% glucose during the holding period. Mortality was recorded 24 h post-exposure.

### Dissections to assess parity status and estimate the number of gonotrophic cycles

Following insecticide bioassays, live and dead mosquitoes 24 h post-exposure, were immediately dissected (n = 1577). The dissections were done under the stereo-microscope and the dissected ovaries were observed under a compound microscope for the presence or absence of the coiled tracheolar skeins indicating nulliparous or parous status respectively [33, 34]. Since studies on landscape distribution of the biological age of mosquitoes can be affected by the methods used for mosquito collection, an additional cohort of mosquitoes collected using the CDC light traps (n = 560) and the DN-Mini trap (n = 78), and not tested for resistance were dissected for more detailed identification of the ovariole dilations to determine how many times the mosquito had laid eggs (i.e. the number of gonotrophic cycles) [34, 35]. The results of the dissections were recorded by distance from the aquatic habitat(s) and method of collection.

### Molecular identification of the mosquitoes

A sub-sample of the tested mosquitoes (at least 10% from each replicate, n = 409) was packed in micro-centrifuge tubes containing silica desiccant. Sibling species of *An. funestus* were identified by PCR using nucleic acid material extracted from the legs, to screen for species-specific nucleotide sequences (internal transcribed spacer 2) in the ribosomal DNA (rDNA) [36].

### Data analysis

The percentage mortality of mosquitoes was calculated as a fraction of the total number exposed and interpreted according to the WHO guidelines [32]. Since no control mortalities exceeded 5%, observed but not corrected mortalities are reported. The mosquitoes were considered susceptible if mortality was $\ge$98%, resistant, if mortality was < 90% or possibly resistant and requiring additional tests if mortality ranged between 90% and 97% [32]. The mean proportions of parous mosquitoes at different distances were compared using the analysis of variance (ANOVA). To determine which specific distance pairs were significantly different, Tukey’s post hoc test was applied.

Additional analysis was done using statistical software, R-Software version 3.6.0 [37]. A generalized linear mixed effect model (GLMM) with binomial distribution and logit link function was used to assess the proportion of mosquitoes that died 24 h post-insecticide exposure at different age groups and distances. Age and distance were added as fixed factors, whereas replicate was added as a random factor. The nearest distance to the habitats (50–100 m) and the youngest age class of mosquitoes (3–5 days) were considered as references for these analyses. Odd ratios with their corresponding 95% confidence intervals were reported. Statistical significance was considered when the p-value is less than 0.05.

## Results

### Relationship between insecticide susceptibility and distance from aquatic habitats

**Fig. 2:**
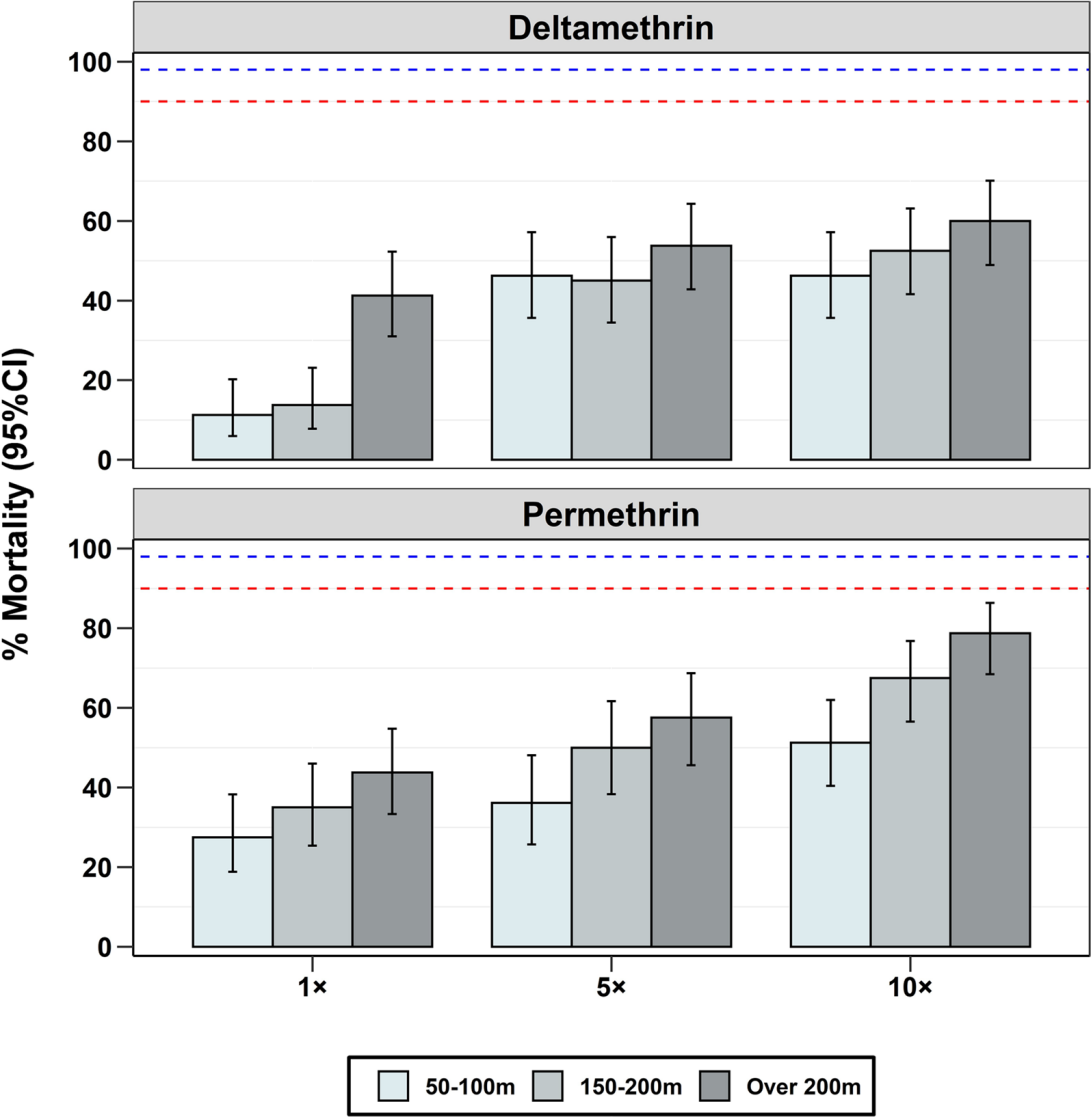
Percentage mortality in *An. funestus* mosquitoes, collected at different distances from the aquatic habitats and exposed to deltamethrin or permethrin. The red-dotted lines represent 90% mortality and the blue-dotted lines represent 98% mortality

Mosquitoes collected in different houses were resistant to both deltamethrin and permethrin at the diagnostic doses (1×) but the percentage of mortality varied by distance from the aquatic habitats (Fig. 2). The mosquitoes collected farthest from the habitats showed significantly higher mortality than those collected nearest to the habitats (Table 1). A similar trend was observed when the mosquitoes were exposed to 5× and 10× the diagnostic doses of permethrin but not deltamethrin, with mosquitoes from the nearest distances showing the lowest mortality and those at the farthest distance showing the highest mortality. Overall, there was lower mortality in tests against deltamethrin compared to permethrin (Fig. 2). Further analysis showed that the association of 24-h mortality with distance was statistically significant for deltamethrin only at the diagnostic dose, and for permethrin at all doses (Table 1).

**Table 1:** Summary of 24-hour mortality of *Anopheles funestus* mosquitoes in tests of adults collected at different distances from aquatic habitats and exposed to deltamethrin or permethrin with or without the synergist, piperonyl butoxide (PBO)

Insecticide	Insecticide dose	Distance (m)	Mean mortality (95% CI)	OR (95% CI)	p-value
		50–100	11% (5.96–20.23)	Ref.	Ref.
	1×	150–200	14% (7.78–23.15)	1.26 (0.49–3.22)	0.633
		> 200	41% (31.03–52.29)	5.54 (2.43–12.63)	< 0.001*
		50–100	46% (35.67–57.18)	Ref.	Ref.
Deltamethrin	5×	150–200	45% (34.50–55.97)	0.95 (0.51–1.77)	0.873
		> 200	54% (42.82–64.33)	1.35 (0.73–2.51)	0.343
		50–100	46% (35.67–57.18)	Ref.	Ref.
	10×	150–200	53% (41.61–63.16)	1.28 (0.69–2.39)	0.429
		> 200	60% (48.95–70.11)	1.74 (0.93–3.26)	0.082
		50–100	28% (18.84–38.26)	Ref.	Ref.
	1×	150–200	35% (25.38–46.02)	1.42 (0.72–2.78)	0.307
		> 200	44% (33.34–54.75)	2.05 (1.06–3.97)	0.033*
		50–100	36% (25.68–48.10)	Ref.	Ref.
Permethrin	5×	150–200	50% (38.31–61.70)	1.76 (0.93–3.31)	0.080
		> 200	58% (45.60–68.70)	2.38 (1.26–4.49)	0.008*
		50–100	51% (40.41–61.97)	Ref.	Ref
	10×	150–200	68% (56.54–76.83)	1.98 (1.04–3.75)	0.037*
		> 200	79% (68.44–86.36)	3.53 (1.76–7.04)	< 0.001*
		50–100	82% (69.85–89.55)	Ref.	Ref.
Permethrin + PBO	PBO	150–200	93% (83.54–97.48)	3.14 (0.94–10.51)	0.063
		> 200	92% (81.49–96.49)	2.47 (0.80–7.61)	0.115
		50–100	85% (73.61–92.01)	Ref.	Ref.
Deltamethrin + PBO	PBO	150–200	98% (89.10–99.77)	10.41 (1.28–85.00)	0.029*
		> 200	95% (85.61–98.38)	3.35 (0.86–13.07)	0.081

OR: Odds ratio; CI: Confidence interval; Insecticide dose: fold increase on standard dose; Reference distance (Ref): 50-100 m; * denotes statistical significance at p = 0.05, relative to the reference distance

**Fig. 3:**
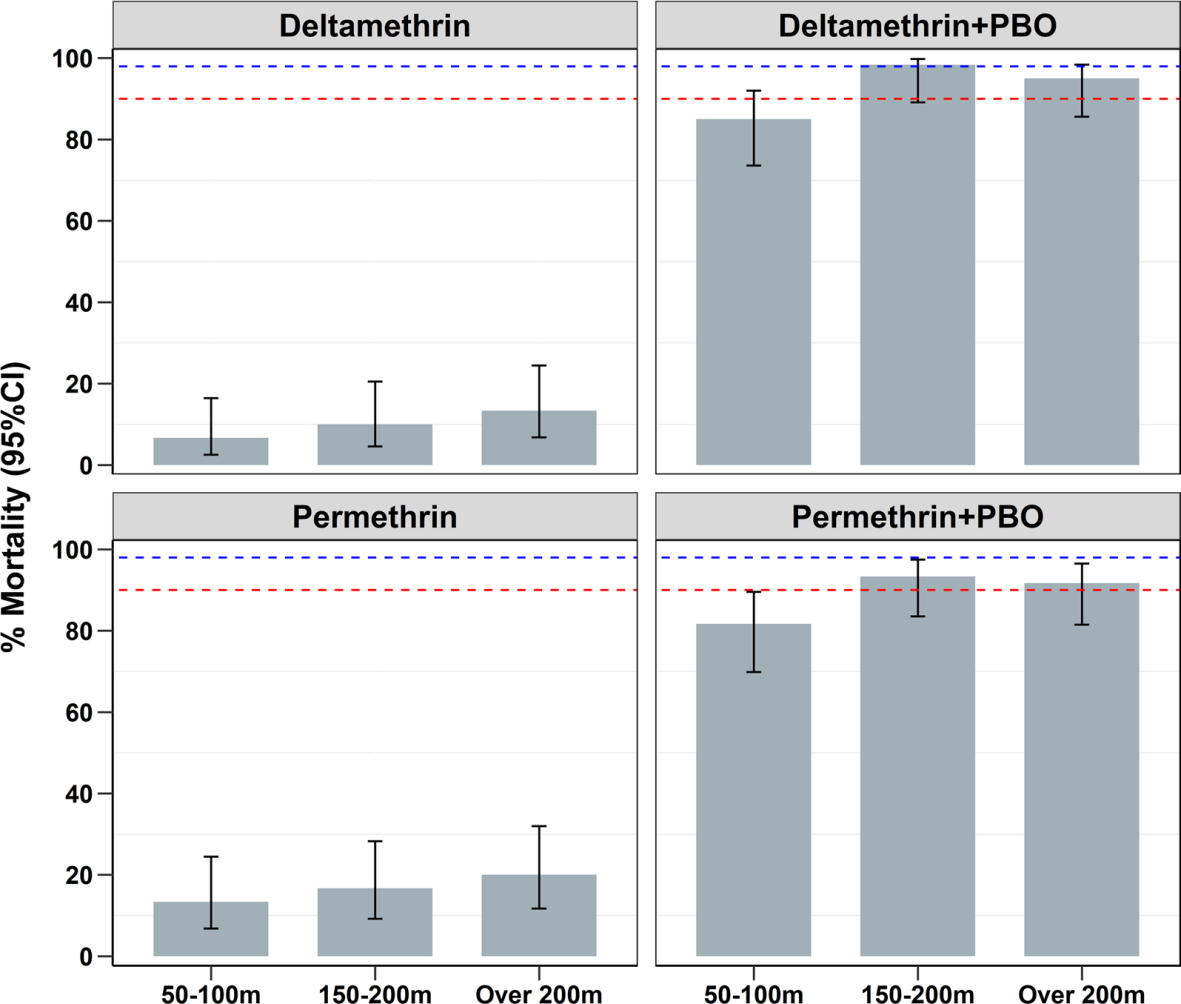
Percentage mortality in *An. funestus* mosquitoes, collected at different distances from the aquatic habitats and exposed to either the candidate pyrethroids alone or PBO followed by pyrethroids. The red-dotted lines represent 90% mortality and the blue-dotted lines represent 98% mortality

Pre-exposure to the synergist, PBO, significantly increased the potency of the candidate pyrethroids against the resistant mosquitoes, achieving more than 80% mortality at all distances when tested using the diagnostic doses (Fig. 3). However, the association of 24-h mortality with distance in these PBO tests was statistically significant only for deltamethrin (Table 1).

### Relationship between biological age of mosquitoes and distance from aquatic habitats

**Fig. 4:**
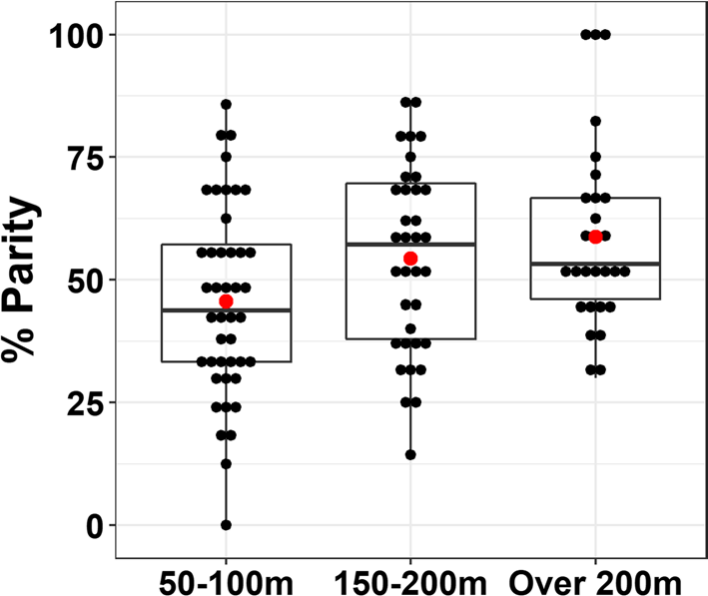
The parity percentages of *An. funestus* mosquitoes, collected at different distances and pre-exposed to insecticide. Red dots indicate the means

**Fig. 5:**
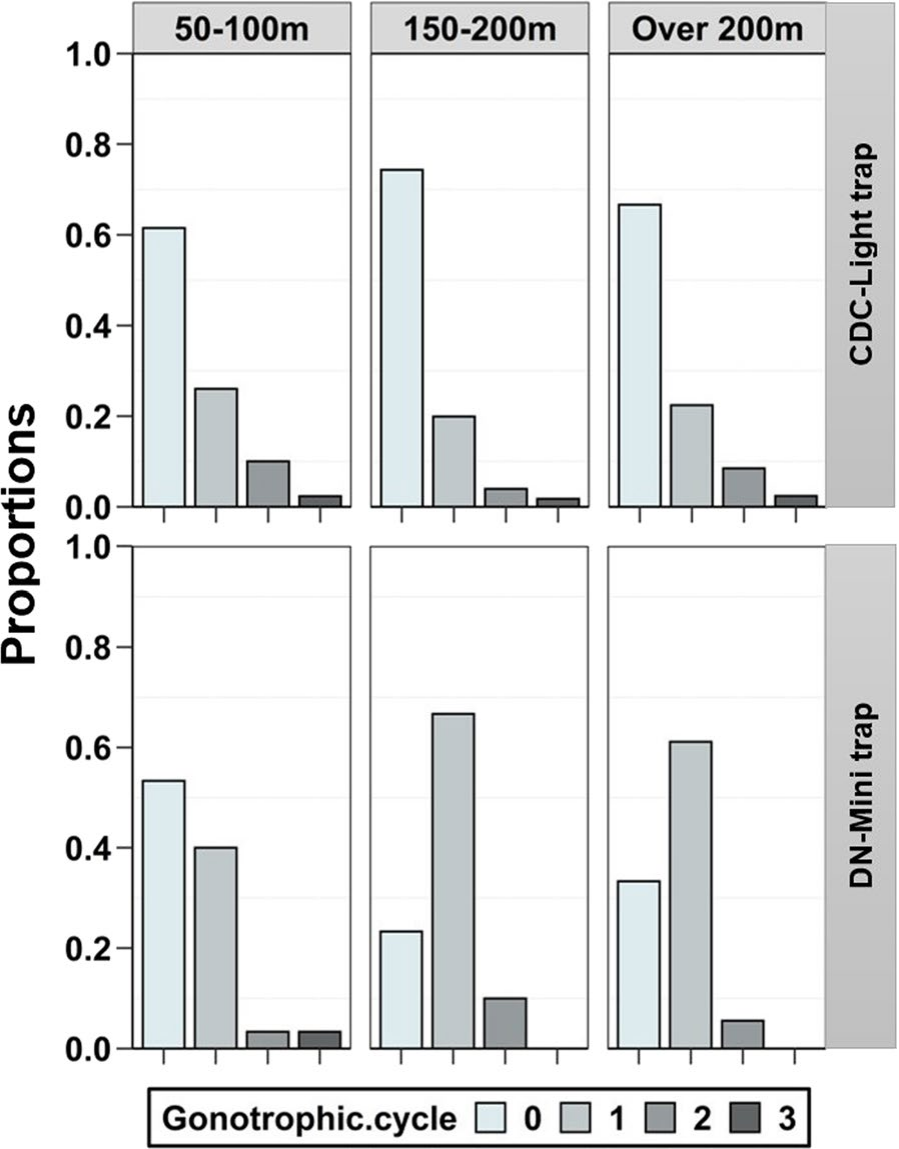
Proportions of mosquitoes with different numbers of gonotrophic cycles, among those collected by either CDC light traps (top panel) or the miniaturized double net (DN-Mini) trap (bottom panel) at different distances

A total of 1397 female *An. funestus* were dissected from the first cohort. Although the overall difference was statistically significant (F = 4.38, p = 0.015), only the mosquitoes collected by CDC light traps farthest from aquatic habitats (over 200 m) had significantly greater proportions of parous mosquitoes compared to those collected nearest to the habitats (50–100 m) (p = 0.017, Fig. 4). Some cohorts of mosquitoes collected beyond 200 m were 100% parous. When the additional cohort of mosquitoes collected by the light traps was assessed for the number of gonotrophic cycles (n = 560), the majority were found to be nulliparous; and those with multiple gonotrophic cycles were equally distributed over the distances (50–100 m, 150–200 m, over 200 m) (Fig. 5). On the other hand, among the mosquitoes collected by DN-Mini (n = 78), the proportion that was nulliparous dropped while the proportion with multiple gonotrophic cycles was slightly higher at 150–200 m and $>$200 m distances compared to 50–100 m (Fig. 5).

### Relationship between insecticide susceptibility and chronological age of mosquitoes

**Fig. 6:**
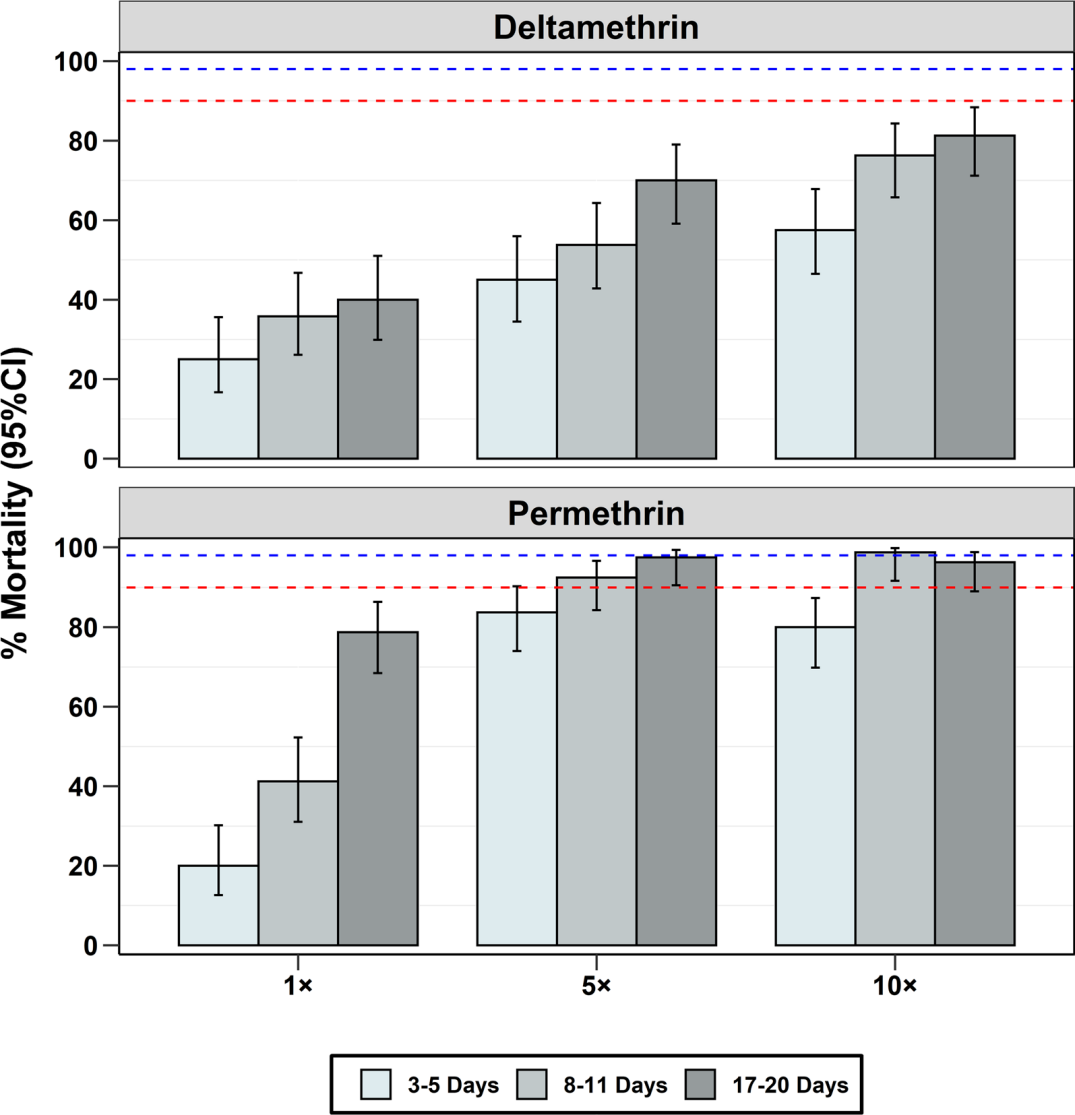
Percentage mortality in different age cohorts of emergent *An. funestus* mosquitoes, collected as larvae and exposed to deltamethrin or permethrin. The red-dotted line represents 90% mortality, the blue-dotted line represents 98% mortality

For both pyrethroids, at the diagnostic dose (1×), the percentage of mortality increased with age, suggesting that the older *An. funestus* were more susceptible than younger ones from the same locations (Fig. 6, Table 2). Similarly, in the intensity bioassays conducted with 5× or 10× the diagnostic doses, the levels of resistance varied by age, but mortality significantly increased, reaching 99% for permethrin and 81% for deltamethrin (Fig. 6). Further analysis showed that the association of 24-hour mortality with mosquito age was statistically significant for both deltamethrin and permethrin, and at all doses (Table 2).

**Table 2:** Summary of 24-hour mortality of *Anopheles funestus* mosquitoes collected as larvae and exposed to pyrethroids (deltamethrin or permethrin) at different age classes (3–5, 8–11 or 17–20 days old)

Insecticide	Insecticide dose	Age (days)	Mean mortality (95%CI)	OR (95% CI)	p–value
		3–5	25% (16.73–53.60)	Ref.	Ref.
	1×	8–11	36% (26.15–46.76)	1.67 (0.85–3.30)	0.138
		17–20	40% (29.89–51.05)	2.00 (1.02–3.93)	0.044*
		3–5	45% (34.50–55.97)	Ref.	Ref.
Deltamethrin	5×	8–11	54% (42.82–64.33)	1.42 (0.76–2.65)	0.269
		17–20	70% (59.12–79.01)	2.85 (1.49–5.46)	0.002*
		3–5	58% (46.48–67.82)	Ref.	Ref.
	10×	8–11	76% (65.73–84.31)	2.37 (1.20–4.68)	0.013*
		17–20	81% (71.20–88.37)	3.35 (1.57–6.55)	0.001*
		3–5	20% (12.63–30.19)	Ref.	Ref.
	1×	8–11	41% (31.03–52.29)	2.81 (1.39–3.69)	0.004*
		17–20	79% (68.44–86.36)	14.82 (6.89–31.89)	< 0.001*
		3–5	84% (74.00–90.32)	Ref.	Ref.
Permethrin	5×	8–11	93% (84.30–96.59)	2.39 (0.86–6.65)	0.094
		17–20	98% (90.55–99.37)	7.57 (1.65–34.74)	0.009*
		3–5	80% (69.81–87.37)	Ref.	Ref.
	10×	8–11	99% (91.66–99.82)	19.75 (2.55–25.95)	0.004*
		17–20	96% (89.01–98.79)	6.42 (1.79–23.01)	0.004*

OR: Odds ratio; CI: Confidence interval; Insecticide dose: fold increase on standard dose; Reference age (Ref): 3–5 days old; * denotes statistical significance at p = 0.05, relative to the reference age

### Molecular identification of the mosquitoes

A total of 409 *An. funestus* mosquitoes were tested by PCR to identify sibling species in the *An. funestus* group, including 60% (n = 245) from mosquitoes collected as adults; and 40% (n = 164) of the adults emerging from larval collections. The amplification rate for the adult collected mosquitoes was 95% (n = 123), and all of them (100%) were identified as *An. funestus sensu stricto* (*s.s.*). On the other hand, the amplification rate for those collected as larvae was 90% (n = 148), of which 99.3% (n = 147) were *An. funestus s.s*, the remaining one specimen being *Anopheles rivulorum*.

## Discussion

To improve surveillance and control of malaria in endemic Africa, there is need for greater understanding of both landscape and local variations in the risk of transmission [7, 38]. Data on population distribution, age-structure and susceptibility of malaria vectors are particularly crucial for analysing the local risk patterns, designing fine-scale targets of interventions and selection of appropriate interventions. This study demonstrated a positive correlation between the mortality of *An. funestus* females exposed to the pyrethroid insecticides and the distance between the sites where these mosquitoes were collected and the nearest aquatic habitats. A positive correlation was observed in tests with the diagnostic dose of insecticides as well as in tests with the five and ten-fold increases in the diagnostic dose. The highest mortalities were obtained among mosquitoes farthest from the aquatic habitats, nearest to the centre of the village, and among the oldest mosquito cohorts. This suggests that insecticidal interventions may have differential impact dependent on distance from the main mosquito habitats.

Mathematical predictions by Smith et al. initially showed that human-biting densities would be greatest near breeding sites where adult mosquitoes emerge, even as the proportions old enough to transmit malaria would be found far from the habitats [7]. An important question, therefore, was how the insecticide resistance profiles influence this system, and whether these potentially infectious adults would also be the most susceptible as suggested by the first experiments in this study. This study has provided a unique opportunity for a concurrent analysis of the age distribution and insecticide susceptibility of malaria vectors over the same distances, thus enabling improved understanding of these variations and their potential epidemiological implications. The proportion of parous females was greatest among the mosquitoes collected farthest from the aquatic habitats, compared to those collected nearest to the aquatic habitats. These findings validate previous studies using the mark-release and recapture techniques, where older female *Anopheles* mosquitoes were collected at the farthest distance, over 600 m from a point of release and survived over 17 days in the wild [15]. Such non-random age distribution of mosquito populations could have a direct impact on the disease transmission risk [6, 7, 9].

Less than 30% of the mosquitoes caught by the CDC had two or more gonotrophic cycles, and the maximum number of cycles was three. The low proportions of old mosquitoes in light traps may reflect the tendency of CDC light traps to catch mostly nulliparous mosquitos in such settings [39, 40]. Secondly it may be due to the fact that only live or freshly dead mosquitoes were dissected, yet parous mosquitoes can be more likely to die during collection [39]. Interestingly, when the DN-Mini trap was used, the proportion of nulliparous mosquitoes decreased (relative to the CDC light trap collections), and there were significant proportions with at least one or two gonotrophic cycles; and a slight increase in proportions with multiple gonotrophic cycles at distances far from the habitats. A related lesson here is that trapping methods may yield biased catches requiring careful interpretation in a broader context [30, 41].

In the tests using mosquitoes collected as larvae from the field sites, the age-synchronized females also showed positive correlations between the chronological age classes and the insecticide potency. The youngest *An. funestus* (3–5 days old) were the most strongly resistant to the candidate insecticides, while the oldest mosquitoes (17–20 days old) were the least resistant. These findings suggest that despite widespread pyrethroid resistance, older mosquitoes may remain substantially susceptible and that insecticidal interventions may retain some degree of efficacy in the communities. The findings by Collins et al., using the wild population, the resistant sample were more likely to be nulliparous compared to the susceptible samples [42]. This is important since it is the older mosquitoes that are responsible for parasite transmission as mosquitoes need approximately 2 weeks for parasite maturity in their bodies [7, 17]. Future research should, therefore, investigate whether this demographic of mosquitoes can or should be selectively targeted and how such a strategy could impact malaria control. Several studies have indeed demonstrated age-related variations in insecticide resistance, in mosquito species other than *An. funestus* [18–20, 43–45], but practical applications of this knowledge remain limited. This might be explained by the fact that insecticide metabolism by mosquitoes decreases with age due to reduced active enzymes [18, 46–48]. Other factors like blood-feeding and larval nutrition may also play a role in insecticide-induced mortality [45, 49].

The tests where mosquitoes were pre-exposed to PBO showed potential significant involvement of mixed-function oxidases in the resistance. Overall, the potency of pyrethroids and mortalities significantly increased after the PBO exposures, reaching between 82% and 98%. The lack of full recovery in some mosquitoes may suggest that additional mechanisms may be partially involved in a resistance profile of the local *An. funestus* mosquitoes. It is reasonable to assume that since the pyrethroid resistance in these *An. funestus* populations were broadly metabolic, the associated detoxifying enzymes may also be differentially expressed by age. However, when all the PBO test data were analysed together, there was no significant correlation between the distances (a proxy for population age structure) and the performance of PBO. These differences have been observed in previous studies and the involvement of the enzymes in these age-related differences remain poorly understood. For example, Christian et al., who analysed mosquitoes aged 3 to 30 days, concluded that two resistance-related genes, CYP6P9 and CYP6P13 were most highly expressed at 10 days of age, but that there was generally no correlation between the expression of these genes and mosquito susceptibility to permethrin [43]. On the other hand, Hazelton et al., who measured the activity of glutathione-s-transferases in mosquitoes during growth, maturity and senescence phases concluded that the enzyme activities generally declined with age [48]. Taken together, the results of this study and the previous studies indicate the need to further investigate the differential expression of resistance genes in different mosquito species and in different settings.

Despite the broad success of this study in analysing the correlations between biological ages, distance from aquatic habitats and pyrethroid resistance status of *An. funestus* mosquitoes, there were a few limitations. Firstly, as previously described by Charlwood et al., the older mosquitoes are more likely to die during collection when the CDC-LT is used during specimen collection [39]. This might have marginally confounded the age evaluation of both insecticide pre-exposed and non-exposed cohorts. However, age synchronised, larval collected mosquitoes showed the same trend as the mortality of mosquitoes increased with the increased age and concentration. Thus, these results could still be considered informative. Secondly, fewer mosquitoes from the farthest distance (over 200 m) were dissected compared to the other distances as most of them were susceptible to the insecticide, and were therefore dead and too dry to be dissected. The effect of insecticide pre-exposure on dissection was rectified by the additional collections done using both CDC light traps and DN-Mini. Lastly, due to the difficulties of collecting *An. funestus* at the larval stage, the PBO pre-exposure tests were done only on the adult collections but not on the age-synchronized mosquitoes collected as larvae.

## Conclusion

In south-eastern Tanzania, where *An. funestus* predominates malaria transmission, the older mosquitoes are more likely to be found farthest from the aquatic habitats and near the centre of the villages, and are also less resistant to pyrethroid insecticides. Similarly, in the age synchronized mosquitoes, the oldest cohorts were the least resistant to the candidate pyrethroids. These findings provide new insights into how the insecticide-based interventions could still provide modest levels of protection against malaria in the communities irrespective of the strong insecticide resistance currently reported. Additionally, the findings also imply that greater impact may be achieved if vector control methods were spatially targeted at a fine-scale, in ways that exploit the observed variations in mosquito age and susceptibility to insecticides. While the actual configuration of such a strategy remains to be determined, it could enable a more effective onslaught against the older populations of malaria vectors, which drive pathogen transmission and are more readily controlled by the insecticides.

## Data Availability

Data are available under the Ifakara Health Institute data sharing policy upon request.

## Abbreviations

ITS2: internal transcribed spacer 2 region
IHI: Ifakara Health Institute
IRB: Institutional Review Board
LLINs: Long-lasting insecticidal nets
NIMR: National Institute for Medical Research
CDC: Centre for disease control and prevention
PBO: Piperonyl butoxide
IRS: Indoor residual spraying
ITNs: Insecticide-treated bed nets
WHO: World Health Organizations
PCR: Polymerase chain reaction
DNA: Deoxyribonucleic acid
ANOVA: Analysis of variance
